# Co-dependence of HTLV-1 p12 and p8 Functions in Virus Persistence

**DOI:** 10.1371/journal.ppat.1004454

**Published:** 2014-11-06

**Authors:** Cynthia A. Pise-Masison, Maria Fernanda de Castro-Amarante, Yoshimi Enose-Akahata, R. Cody Buchmann, Claudio Fenizia, Robyn Washington Parks, Dustin Edwards, Martina Fiocchi, Luiz Carlos Alcantara, Izabela Bialuk, Jhanelle Graham, Jean-Claude Walser, Katherine McKinnon, Bernardo Galvão-Castro, Antoine Gessain, David Venzon, Steven Jacobson, Genoveffa Franchini

**Affiliations:** 1 Animal Models and Retroviral Vaccines Section, National Cancer Institute, Bethesda, Maryland, United States of America; 2 Viral Immunology Section, Neuroimmunology Branch, National Institute of Neurological Disorders and Stroke, Bethesda, Maryland, United States of America; 3 Oswaldo Cruz Foundation Salvador, Bahia, Brazil; 4 Department of General and Experimental Pathology, Medical University in Białystok, Białystok, Poland; 5 Evolutionary Biology, Genetic Diversity Centre, University of Basel, Basel, Switzerland; 6 Vaccine Branch Flow Cytometry Core Laboratory, National Cancer Institute, National Institutes of Health, Bethesda, Maryland, United States of America; 7 Unité d'Epidémiologie et Physiopathologie des Virus Oncogènes, Département de Virologie, Batiment Lwoff, Institut Pasteur, Paris, France; 8 Biostatistics and Data Management Section, National Cancer Institute, Bethesda, Maryland, United States of America; Imperial College London, United Kingdom

## Abstract

HTLV-1 *orf-I* is linked to immune evasion, viral replication and persistence. Examining the *orf-I* sequence of 160 HTLV-1-infected individuals; we found polymorphism of *orf-I* that alters the relative amounts of p12 and its cleavage product p8. Three groups were identified on the basis of p12 and p8 expression: predominantly p12, predominantly p8 and balanced expression of p12 and p8. We found a significant association between balanced expression of p12 and p8 with high viral DNA loads, a correlate of disease development. To determine the individual roles of p12 and p8 in viral persistence, we constructed infectious molecular clones expressing p12 and p8 (D26), predominantly p12 (G29S) or predominantly p8 (N26). As we previously showed, cells expressing N26 had a higher level of virus transmission *in vitro*. However, when inoculated into Rhesus macaques, cells producing N26 virus caused only a partial seroconversion in 3 of 4 animals and only 1 of those animals was HTLV-1 DNA positive by PCR. None of the animals exposed to G29S virus seroconverted or had detectable viral DNA. In contrast, 3 of 4 animals exposed to D26 virus seroconverted and were HTLV-1 positive by PCR. *In vitro* studies in THP-1 cells suggested that expression of p8 was sufficient for productive infection of monocytes. Since *orf-I* plays a role in T-cell activation and recognition; we compared the CTL response elicited by CD4^+^ T-cells infected with the different HTLV-1 clones. Although supernatant p19 levels and viral DNA loads for all four infected lines were similar, a significant difference in Tax-specific HLA.A2-restricted killing was observed. Cells infected with Orf-I-knockout virus (12KO), G29S or N26 were killed by CTLs, whereas cells infected with D26 virus were resistant to CTL killing. These results indicate that efficient viral persistence and spread require the combined functions of p12 and p8.

## Author Summary

HTLV-1 persists despite a vigorous host immune response. We found that polymorphism of HTLV-1 *orf-I* alter the relative amounts of the p12 precursor and its cleavage product p8, and is associated with differences in blood virus levels in humans, a correlate of disease risk. Reverse genetics in 160 HTLV-1 infected individuals demonstrated that equivalent levels of p8 and p12 are associated with high virus levels and, accordingly, genetically engineered HTLV-1s that express either predominantly p12 or p8 are poorly infectious in macaques. We found that expression of p8 is sufficient for productive infection of monocytes. Expression of either p12 alone or p8 alone is insufficient to protect infected cells from MHC-class-I restricted CTL killing. However, the balanced expression of both provides resistance of infected cells to CTL killing. Together, our findings provide the rationale to explore novel approaches to target the cleavage of the p12 protein, an essential step for viral infectivity and persistence.

## Introduction

**Figure 1:**
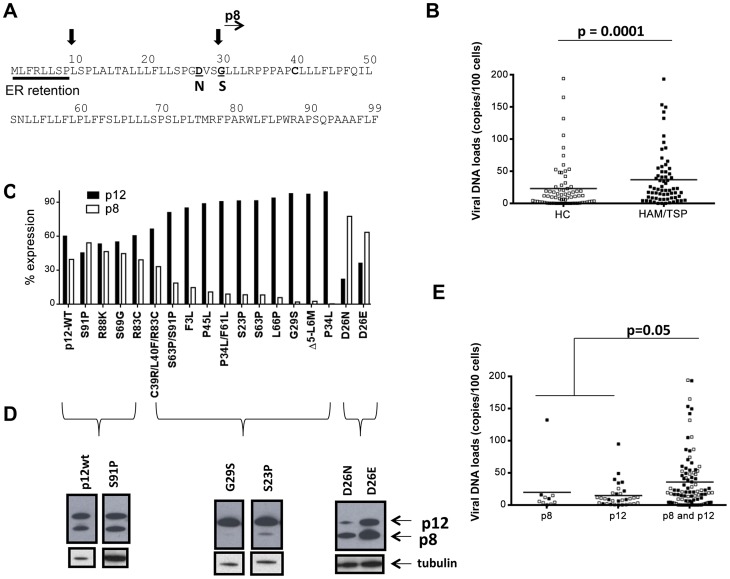
Analysis of *orf-I* from the PBMCs of HTLV-1 infected individuals. (A) Schematic diagram of the Orf-I protein. The non-canonical endoplasmic reticulum (ER) retention sequence is underlined by a solid bar. Black arrows indicate the putative cleavage sites, as well as the start of the p8 isoform. Mutations which identify cleavage variants at position 26 and 29 are indicated in bold below the sequence. (B) Comparison of viral DNA levels in PBMCs from HTLV-1 infected individuals by disease association, HC: healthy carrier (open symbols) and HAM/TSP: HTLV-1 associated myelopathy/tropical spastic paraparesis (filled symbols). The data from 70 healthy carriers (HC) (n = 70) and 66 HAM/TSP individuals (n = 66) were analyzed using the Mann-Whitney Test stratified by disease status. The statistically significant difference is marked with the *p* value. The horizontal lines represent the mean viral DNA load. (C) Cloned *orf-I* cDNA constructs were transfected into 293T-cells and protein expression analyzed 48 hours after transfection. The density of p12 and p8 bands was measured using AlphaView Software on an AlphaImager (ProteinSimple, San Leandro, CA). Expression of p12 and p8 were added to give 100% expression. The percent of total Orf-I expression for each clone was graphed. The black bars represent the percentage of p12 expressed and the lighter bar represents the percentage of p8 expressed. The clone is indicated at the bottom of the graph. Expression patterns for each clone were examined in independent transfection experiments where n = 20 for D26, n = 8 for G29S; P45L, n = 7 for P34L/F61L, n = 6 for S69G; S23P; S63P; D26E; P34L, n = 5 for C39R/L40F/R83C; F3L; L66P; Δ5-L6M, n = 4 for R83C, D26N, n = 3 for S91P, n = 2 for R88K; S63P/S91P. The expression patterns could be divided into three groups: p12 and p8, p12 mainly (p12) or p8 mainly (p8). (D) Representative western blot analysis of cell lysates for Orf-I expression, using anti-HA (upper panel) or a loading control (anti-tubulin, lower panel) was performed. Amino acid changes are indicated above each lane. The p12 or p8 isoform is indicated by arrows at the right. (E) Viral DNA levels in PBMCs from individuals with the indicated *orf-1* gene expression patterns are indicated in the x-axis. The data obtained was a total of 136 individuals using the same assay (n = 10 individuals with mainly p8 expression, n = 32 individuals with mainly p12 expression and n = 94 individuals with similar p12 and p8 expression) and analyzed by an exact Wilcoxon rank sum test stratified by disease status. The horizontal lines represent the mean viral DNA levels. The open symbols identify healthy carriers and the filled symbols HAM/TSP patients. The statistical significance is indicated by the *p* value.

HTLV-1 causes Adult T-cell Leukemia/Lymphoma (ATLL) [1], [2] or HTLV-1 Associated Myelopathy/Tropical Spastic Paraparesis (HAM/TSP) in approximately 2–3% of the 15–20 million individuals infected by the virus worldwide [3], [4]. HTLV-1 persists in the host despite a vigorous cellular and antibody response, suggesting that the virus has developed effective mechanisms to counteract host immune surveillance [5]. The HTLV-1 open reading frame-I (*orf-I*) protein products p12 and p8 increase NFAT activity [6], [7], STAT-5 transcriptional activity and IL-2 production [8]–[10] in T-cells. In addition, they cause down-regulation of ICAM-1 and -2, but not ICAM-3 surface expression, allowing escape of infected cells from NK cell killing [11]. The p12 protein precursor is processed by proteolytic cleavage that removes a non-canonical endoplasmic reticulum (ER) retention/retrieval signal at its amino-terminus to yield p8 (Figure 1A) [12], [13]. The p8 protein traffics to the cell surface, is recruited to the immunological synapse following T-cell receptor (TCR) ligation, and down-regulates TCR proximal signaling [14]. In addition, p8 increases cell adhesion and virus transmission and is transferred to neighboring cells via cellular conduits [15]. Both p8 and p12 can form homo- or hetero-dimers through a highly conserved single cysteine (position 39) or are palmitoylated and remain monomeric (Figure 1A) [16], [17]. *Orf-I* knockout viruses are not infectious in non-human primates [18], suggesting the importance of *orf-I* in human infection. Here, studying a cohort of 160 HTLV-1 infected individuals, using an experimental model of macaque infection and using *in vitro* relevant models of HTLV-1 infection, we demonstrate that natural mutations within *orf-I* can affect the relative amounts of p12 and p8, which in turn, correlate with viral DNA levels in blood, the best predictor of risk for the development of HAM/TSP or ATLL [19]–[22]. In addition, we demonstrate that both proteins are essential for the *in vitro* resistance to cytotoxic T-lymphocyte (CTL) killing of HTLV-1 infected cells.

## Results

### HTLV-1 *orf-I* in humans

Analysis of *orf-I* was performed on 160 HTLV-1 infected individuals from various geographical areas (Table 1), 79 had HAM/TSP and 81 were carriers. Genomic DNA was isolated from patient PBMCs and used to quantify the viral DNA load and for studies on the *orf-I* gene. As expected, individuals with HAM/TSP had significantly (p = 0.0001) higher PBMC viral DNA loads than carriers (Figure 1B). We obtained DNA sequences for a total of 834 clones from these patients and compared them to our reference *orf-I* cDNA [13] and found 216 variants (i.e., one or more nucleotide changes compared to the consensus sequence). One hundred thirty of these variants (85%) were unique. The most frequent non-synonymous mutations within the o*rf-I* gene yielded G29S, P34L, S63P, R88K, and S91P amino acid changes. In line with *orf-I* being necessary for infection, none of the approximately 1600 o*rf-I* sequences analyzed had a premature termination codon. We selected 17 non-synonymous mutations based on either their proximity to the cleavage sites or their frequency in humans and inserted them into the reference o*rf-I* cDNA (herein defined as p12WT) and transfected the expression constructs into 293T-cells. The relative amount of p8 and p12, evaluated by Western blot and densitometric scans was calculated as a percentage of total expression from the *orf-I* gene (Figure 1C). A minimum of two up to 20 independent Western blot experiments were performed as indicated for each mutant in the legend of Figure 1C. We observed 3 distinct patterns of expression (Figure 1C and Figure 1D). The first consisted of balanced expression of p8 and p12, as for p12WT. All these mutations were downstream of both cleavage sites (mutants S91P-61% of patients, R88K-10% of patients, S69G-12% of patients and R83C-6% of patients). The second class consisted of predominant expression of p12 (mutants F3L-4% of patients, P45L-9% of patients, S23P-15% of patients, P34L/F61L-23% of patients, S63P-62.5% of patients, L66P-10% of patients and G29S-30% of patients) (Figure 1A). The viral DNA loads for patients with the G29S mutations (Supplemental Table S3) follow the same trend as the overall patient pool in that patients with HAM/TSP had higher viral DNA loads. The third pattern was generated by a rare mutation in position 26 between the two cleavage sites from aspartic acid (D) (present in p12 WT, see Figure 1A) to either asparagine (N) (mutated in 5% of patients) or glutamic acid (E) (mutated in 2% of patients), resulting in the predominant expression of p8 [12].

**Table 1:** HTLV-1 patients.

Region	Clinical Status	Patients Number	Total
South America	HAM/TSP	38	83
	Carrier	45	
Africa	HAM/TSP	3	12
	Carrier	9	
North America and	HAM/TSP	23	40
Caribbean	Carrier	17	
Asia	HAM/TSP	1	2
	Carrier	1	
Unknown origin	HAM/TSP	14	23
	Carrier	9	
**Total**			**160**

The *orf-I* sequence from 160 HTLV-1 infected individuals, from the indicated geographical regions, were evaluated. The patients were grouped by clinical status as either an HTLV-1 carrier or HAM/TSP.

Analysis of the three *orf-I* expression patterns and viral DNA levels in blood revealed significantly higher viral levels in individuals whose cDNA expressed both p8 and p12 (p = 0.05), compared to those that predominantly expressed either p8 or p12 (Figure 1E). No correlation with disease status was observed within this patient cohort.

### p8 and p12 are required for persistent infection of macaques

**Figure 2:**
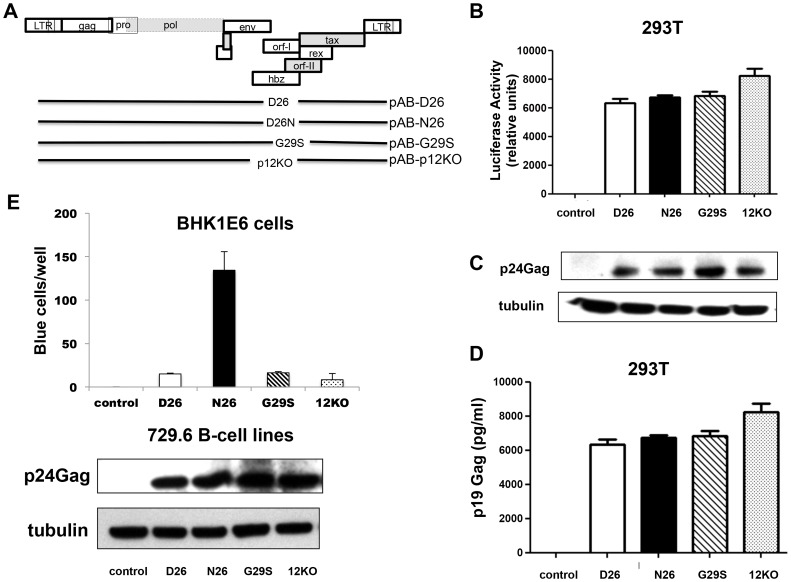
Mutant viruses produce equivalent levels of Gag protein but the virus N26 is transmitted better. (A) The schematic diagram of the HTLV-1 molecular clones indicates the amino acid change in each clone. The initiation codon for Orf-I is mutated in p12KO such that no Orf-I protein is made. The changes did not affect the sequence and/or function of the overlapping pX region genes. Infectious molecular clones or control DNA were co-transfected with an HTLV-1-LTR-luciferase construct and the renilla-luciferase transfection efficiency control into 293T-cells and culture supernatants or protein lysates prepared 48 hours after transfection. (B) The HTLV-1 promoter activity induced by the HTLV-1 mutant was measured by assaying luciferase activity from transfected cell lysates. Luciferase activity for each clone (indicated on the x-axis) from three independent transfection experiments was graphed (n = 3). LTR-luciferase activity was normalized using the transfection efficiency control renilla-luciferase activity. Error bars indicate the standard deviation. (C) Western blot analysis of protein lysates from transfected cells was assayed for intracellular p24Gag expression (top panel) or the loading control, tubulin (bottom panel). (D) Culture supernatants from transfected 293T-cells were collected, spun to remove debris and assayed for p19Gag levels using an HTLV-1 ELISA kit. The values graphed are from three independent experiments (n = 3). (E) Stable producer 729.6 B-cell lines were cloned and used to quantify the transmission of the viral mutants. The 729-HTLV-1-producing cells or parental control cells were co-cultured with BHK1E6 cells and 48 hours later, adherent cells were stained for -galactosidase activity. Graphed is the number of blue cells per well for the indicated clone from three independent wells (n = 3). Error bars indicate standard deviation. By ANOVA and t- test, transmission of WT, D26N and G29S was significantly different than control (p<0.0001). Transmission of D26N was significantly different than WT, G29S and p12KO (p = 0.0007). There was no significant difference among transmission of WT, G29S and p12KO. Western blot analysis for HTLV-1 p24Gag was performed on whole cell extracts from 729-HTLV-1 producing cell lines. The housekeeping gene tubulin is shown for a loading control (lower panels).

To directly assess the requirement of p8 and p12 on viral infectivity and persistence, we engineered the HTLV-1 molecular clone pAB [18], that carries an *orf-I* identical to p12WT designated here as pAB-D26 (Figure 2A). Glycine 29 was substituted with serine to generate pAB-G29S as this mutation impairs cleavage of p12 to p8, resulting predominantly in p12 expression (Figure 1D) [12]–[18]. Because substitution of N or E at position 26, results in predominant expression of p8 (Figure 1D), we generated pAB-N26. Importantly, the mutations introduced in the *orf-I* gene did not alter the amino acid sequences of the *hbz* or *orf-II* genes that overlap with *orf-I*. The isogenic clone pAB-p12KO, mutated at the *orf-I* initiation ATG to eliminate expression of both p8 and p12 (Figure 2A) was used as a control since it is infectious *in vitro* but not infectious *in vivo* [18]. The molecular clones were co-transfected with an HTLV-1-LTR-Lucifease construct into 293T-cells to demonstrate their equivalent ability to produce the Tax protein and activate the viral LTR (Figure 2B). All viruses produced equivalent amounts of intracellular p24Gag (Figure 2C) and extracellular p19Gag (Figure 2D).

We generated stable 729.6 human B-cell lines producing the viral mutants as described [18]. These cell lines were clonal and expressed equivalent levels of intracellular p24Gag and extracellular p19Gag (Figure 2E, lower panels). We observed differences in viral transmission when the cell lines were co-cultured with the reporter cell line, BHK1E6 [23], which contains the β-galactosidase gene under the control of the HTLV-1-LTR promoter. The D26, G29S, or 12KO viruses were transmitted equivalently, but the N26 virus was transmitted 10-fold more efficiently (Figure 2E), consistent with the ability of p8 to increase virus transmission [15].

**Figure 3:**
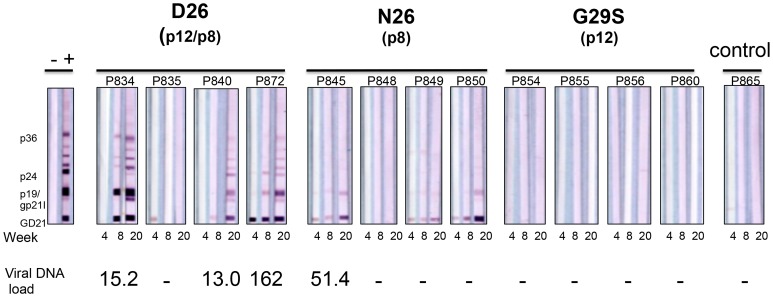
D26, N26, and G29S infectivity in macaques. Sera from inoculated male Rhesus macaques were assayed for reactivity to HTLV-1 antigens. The animal number and inoculation group are indicated above each sample. Indicated below each western blot strip is the time of sera collection. The presence of HTLV-1 viral DNA was measured from PBMC DNA isolated at the designated time points by PCR analysis for HTLV-1 integrase; (-) indicates PCR negative. Viral DNA loads were normalized to the macaque albumin gene and expressed as the number of HTLV-1 viral DNA copies per 10^6^ PBMCs. The value of the viral DNA load provided at the bottom of the figure is the highest measured for the indicated animal.

Whether it is p8, p12, or both that contribute to the requirement of *orf-I* for infection *in vivo* remains unclear [18]. To address this point, we inoculated intravenously the lethally γ-irradiated B-cell lines producing equivalent levels of p19Gag (Supplemental Table S1) from the D26 virus into four macaques, the N26 virus into four macaques and the G29S virus into four macaques. One animal was inoculated with parental uninfected 729.6 cells as a control. Three (P834, P840, P872) of the four animals exposed to the D26 virus became HTLV-1 positive by PCR with viral levels greater than 50 copies per one million cells for at least one time point throughout the study and fully seroconverted for viral antigens (Figure 3). In contrast, only one animal (P845) exposed to the N26 virus was PCR positive for viral DNA and only three animals showed weak reactivity to HTLV-1 antigens. None of the four animals in this study or four animals from a previous study exposed to G29S virus became PCR positive or seroconverted (Figure 3). We verified that the virus in animal P845, infected with N26 virus, retained the mutation at position 26 by cloning and sequencing *orf-I* from its PBMCs. These results suggest that expression of both p12 and p8 is required for efficient HTLV-1 infection and viral persistence. However, they also suggest that p8 may be sufficient for infection and at least partial seroconversion; particularly since none of the eight animals inoculated with virus predominantly expressing p12 seroconverted or had detectable viral DNA.

### p8 is essential for productive infection of monocytes

**Figure 4:**
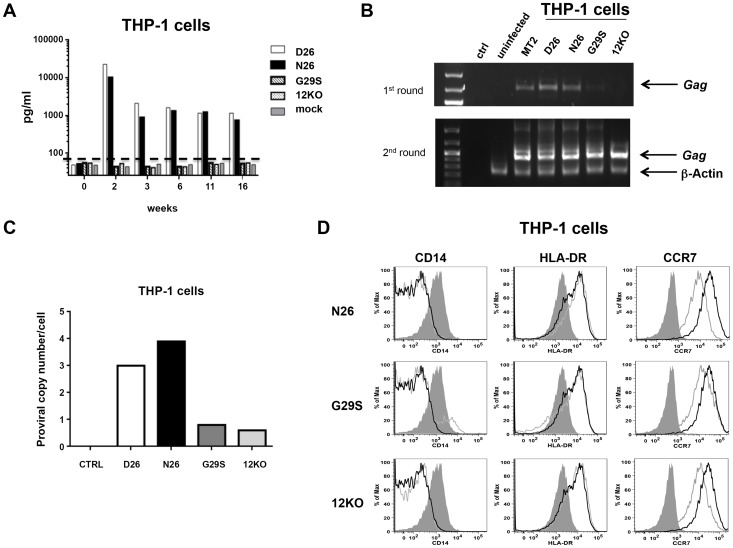
HTLV-1 infection of the monocytic cell line THP-1. (A) THP-1 cells were infected with supernatants from 729-HTLV-1 producing or parental 729.6 cell lines (concentrated by ultracentrifugation). Culture supernatants were monitored by ELISA for p19Gag levels. Graphed is the log scale of p19Gag in picograms per milliliter over a 16 week period for one set of cultures. THP-1 infected cultures: D26 (white bar); N26 (black bar); G29S (slanted bar); 12KO (dotted bar); Mock (gray bar). The dashed line indicates assay background level. (B) PCR analysis was performed on genomic DNA isolated at week 16. The first (upper panel) and second (lower panel) rounds of nested PCR were separated by electrophoresis and stained with ethidium bromide to visualize products for the indicated cell cultures. Arrows designate the *Gag* and the control *β-actin* fragments. (C) The viral DNA copy number for each cell culture at week 18 was determined by quantitative real-time PCR. The human albumin gene was used for normalization. (D) Histogram plots show the phenotype of HTLV-1 infected THP-1 cells for the cell surface monocytic markers: CD14, HLA-DR and CCR7. Each viral mutant (gray line) was compared to the wild-type (D26, un-shaded, black line) and the mock (shaded) infected THP-1 cells.

HTLV-1 infects monocytes and dendritic cells [24]–[26] but the role of infected monocytes to HTLV-1 pathogenesis remains unclear. We have previously demonstrated that the abrogation of *orf-I* expression results in loss of HTLV-1 infectivity of primary monocyte-derived dendritic cells [18] and further that infection of the monocytic cell line THP-1 mirrored results of *ex vivo*, primary dendritic cells [25]. To define the relative contribution of p8, p12, or both to monocyte infection, we exposed the monocytic cell line THP-1 to equivalent amounts of virus as measured by p19Gag from unfiltered cell-free supernatants. Representative cultures are shown (Figure 4). Cultures exposed to D26 or N26 viruses had greater than 10,000 pg/ml of p19Gag in their supernatants at week 2 (Figure 4A) and virus production was maintained up to 16 weeks. In contrast, cultures infected with G29S or 12KO viruses had only background levels of p19Gag, as seen in control cultures (mock-infected with 729.6 culture supernatant). Genomic DNA isolated from the exposed THP-1 cells at week 18 was tested by nested PCR for viral DNA. The level of HTLV-1 DNA detected by PCR was consistent with the level of p19Gag released into the supernatant and was highest in the cultures infected with the D26 and N26 viruses (Figure 4B). Quantitative PCR showed that the D26 and N26 infected cultures contained 3–4 viral DNA copies per cell, while the G29S and 12KO infected cultures contained less than 1 copy per cell (Figure 4C). Interestingly, despite the differences in viral production, all HTLV-1 infected THP-1 cultures displayed down-regulation of CD14 and up-regulation of the activation markers HLA-DR and CCR7 (Figure 4D). These results suggest that p8 expression is necessary and sufficient for productive HTLV-1 infection in monocytes since p8 is expressed in both D26 and N26, but not in G29S and 12KO.

### Evasion of CTL activity requires both p12 and p8

**Figure 5:**
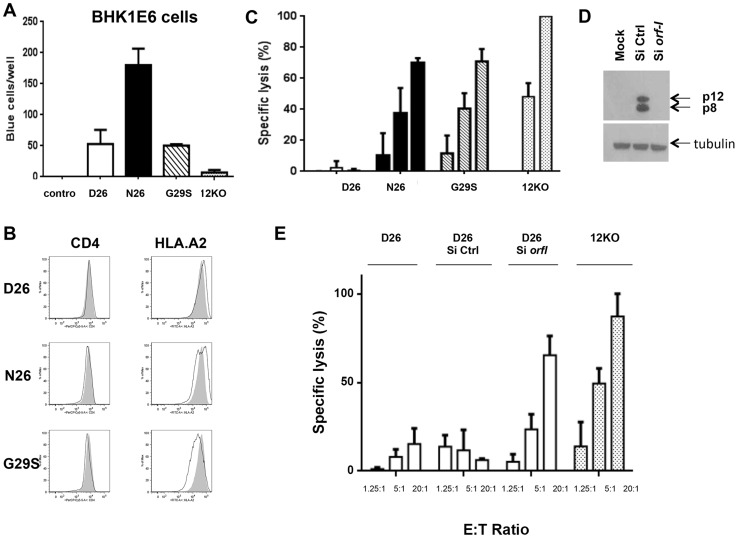
Susceptibility of HTLV-1 producing CD4^+^ cell lines to CTL killing. (A) CD4^+^ T-cells infected with the D26, N26, G29S and 12KO viruses were incubated with BHKE16 indicator cells for 48 hours. Un-infected Jurkat T-cells (control) were used as a negative control. The number of blue cells per well for three independent experiments is graphed (n = 3). Error bars indicate standard deviation. (B) A comparison of the surface expression of CD4 (left panels) and HLA.A2 (right panels) are shown for the indicated virus-infected CD4^+^ T-cells (black line) in comparison to the 12KO CD4^+^ T-cell line (shaded). (C) Cytoxic T-lymphocyte killing assays were done to evaluate specific lysis of CD4^+^ T-cells infected with the D26, N26, G29S and 12KO viruses. A long term HLA.A2 restricted CD8^+^ T-cell line from an HAM/TSP patient was used as the effector cell (see Materials and Methods). Graphed is the percent of specific lysis at varying effector-to-target cell ratios (1.25∶1; 5∶1; 20∶1). The graph represents data from at least two independent experiments done in triplicate (n≥2). Lysis of 12KO at the 20∶1 ratio (highest specific lysis) was set to 100%. All samples were normalized to maximal killing obtained with the 12KO virus. Error bars indicate standard deviation. (D) Western blot analysis of protein lysates from transfected cells was assayed for p12 and p8 expression (top panel) or the loading control, tubulin (bottom panel) to determine the effectiveness of the siRNA constructs. Cells were co-transfected with p12WT cDNA or control expression constructs in the presence or absence of siRNA (Si Ctrl or Si *orf-I*). (E) CD4^+^ D26-infected cells were transfected with siRNA control (Si CTRL) or siRNA to *orf-I* (Si *orf-I*) and used as target cells in CTL killing assays. CTL lysis of the cells at increasing effector-to-target cell ratios is graphed. The graph represents data from at least two independent experiments done in triplicate (n≥2). Error bars indicate standard deviation.

CTLs play an important role in limiting viral replication and spread by recognizing and lysing virally infected cells. The *orf-I* protein products interfere with the normal trafficking of the MHC-class-I molecule and are thought to reduce CTL recognition [27], [28]. To dissect the impact of p12 and/or p8 on CTL responses in the context of the whole virus, we generated immortalized infected CD4^+^ T-cells lines from an HLA.A2 healthy donor that allowed the use of the human CTL clone from a HAM/TSP patient that recognize the HLA.A2 restricted Tax peptide [11]–[19] [29]. The CD4^+^ T-cells were cultured for over a year prior to analysis; viral production, viral DNA copy numbers, and the level of expression of the *orf-I* gene in the infected cultures is summarized in Supplementary Table S2. In line with previous results [15], T-cells producing N26 transmitted virus better than those producing D26, G29S and p12KO (Figure 5A).

We reported previously that *orf-I* expression down-regulates the surface expression of major histocompatibility complex (MHC)-class-I in overexpression models [28]. Interestingly, in here, we observed that the surface expression of HLA.A2 was clearly down-regulated in CD4^+^T-cells that produce the G29S virus indicating that p12 expressed by the virus, down-regulates MHC-class-I in primary human CD4^+^ T-cells (Figure 5B).

Next, we studied whether infection of T-cells with the different viruses affected their susceptibility to CTL killing. The CD4^+^ T-cells infected with D26, N26, G29S and 12KO were loaded with equivalent amounts of the immunodominant Tax [11]–[19] peptides and co-cultured with the CTL clone at various effector-to-target ratios. We observed the highest CTL killing of the 12KO cells, suggesting that the absence of both p8 and p12 makes cells susceptible to CTL killing (Figure 5C). We found reduced killing of cells infected with N26 that predominantly express p8, as well as in G29S that predominantly express p12. Strikingly, we observed nearly complete resistance to CTL killing at all effector-to-target ratios of cells infected with D26 that express a balanced level of p8 and p12 (Figure 5C). The resistance to CTL killing of the D26 infected CD4^+^ T-cells was abrogated by siRNAs targeting the *orf-I* mRNA but not control siRNA (Figure 5D and 5E). These results suggest that balanced expression of p12 and p8 is required to protect HTLV-1 infected cells from CTL killing.

## Discussion

The p12 precursor, encoded by *orf-I*, contains two proteolytic cleavage sites, the first site, between amino acids 9 and 10 and the second site, between amino acids 29 and 30 [12]. The p12 precursor is an ER associated protein and its cleavage removes a non-canonical ER retention/retrieval signal that generates p8, a protein that localizes to the cell surface [12]. Both p8 and p12 interact with the β and γc chains of the interleukin-2 receptor (IL-2R) [10], the heavy chain of the MHC-class-I [28], calreticulin and calnexin [30], and ICAM-1 and ICAM-2 [11]. The p8 protein traffics to lipid rafts, is recruited to the immunologic synapse following T-cell receptor (TCR) ligation where it down-regulates TCR proximal signaling [12] and co-localizes with lymphocyte function-associated antigen-1 (LFA-1), increasing its clustering [15]. The p8 protein also increases T-cell adhesion, the formation of cellular conduits, and HTLV-1 transmission [15]. A novel feature of p8 is its ability to be rapidly transferred from cell-to-cell through cellular conduits [15]. Here, we investigated the specific contribution of each isoform to viral infectivity of T-cells and monocytes *in vitro* and in viral persistence *in vivo*.

We hypothesized, that genetic mutations, which surround the putative cleavage sites, affect the relative levels of p8 and p12 that may have consequences in HTLV-1 infection. By using reverse genetics on samples from HTLV-1 infected individuals, we have identified genetic polymorphisms that affect the efficiency of cleavage of the p12 precursor protein into p8. The first and most frequent group of mutations results in an intermediate efficiency of cleavage that yields an equivalent amount of p8 and p12. This phenotype is associated with productive infection of monocytes and a high viral DNA level in blood that is a correlate of disease development [20]–[22], [31]. The second most frequent phenotype, yields predominantly p12 and affects the ability of the virus to productively infect monocytes. The third rarer phenotype, results in higher levels of p8 and a virus that retains its ability to productively infect monocytes. However, consistent with our studies in macaques and CTL sensitivity, both the second and third phenotypes are associated with low virus DNA levels in blood of naturally infected humans. We found that HLA-DR expression, as well as CD80 and CCR7 were up-regulated in monocyte cultures infected by HTLV-1, even when low or undetectable viral proteins are expressed. The role of infected monocytes to HTLV-1 pathogenesis is unclear. However, HTLV-1 infection of monocytes has been demonstrated [25], [26], [32], [33] and viral infection is associated with an increased frequency of more differentiated monocytes (CD16^bright^) that may spread the virus to tissues [34].

These results suggest that *orf-I* plays a role in viral persistence however, an early study by Furukawa and colleagues [35] found in one HAM/TSP patient a virus with a mutation at the start codon of *orf-I* and that this virus was transmitted in the individual's family. In contrast to our work, Furukawa et al. did not clone the *orf-I* products and assess its expression and stability [35]. In addition, the authors have not ruled out that the *orf-I* gene was expressed in those individuals through alternative splicing. Several groups have shown that cryptic splice sites and donor sites are present in retroviral sequences and that gene products can be produced through alternative splice acceptor/splice donor usage. Thus, although we have not demonstrated an absolute requirement for *orf-I* in HTLV-1 infection in humans, it is clearly required in non-human primates [18]. In addition, the results of this study and that of Furukawa et al. [35], finding only 1 in 304 patients which do not retain *orf-I* expression (0.3%), suggests that *orf-I* expression is likely to provide an advantage in HTLV-1 persistence.

**Figure 6:**
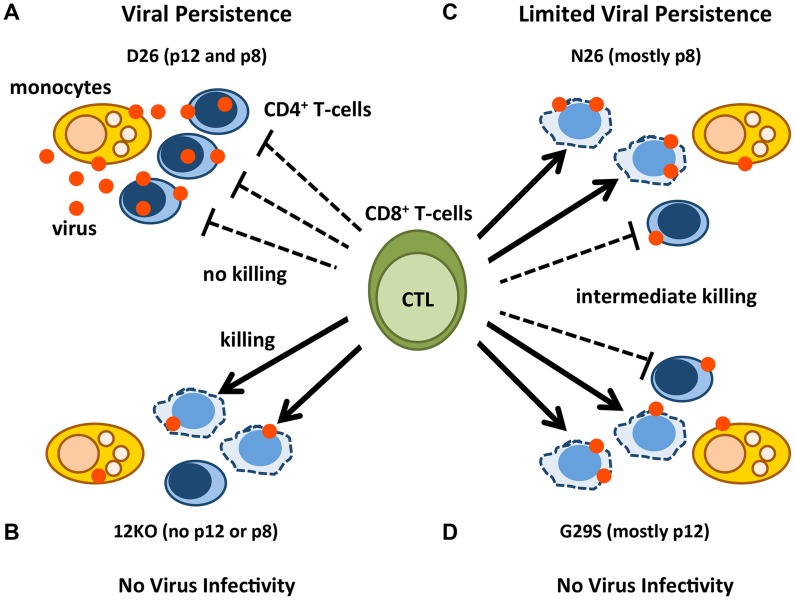
Model of p12 and p8 functions on monocyte, T-cell infection, and their susceptibility to CTL killing. The red dots represent HTLV-1 virions/proteins and the solid arrows represent effective CTL killing of CD4^+^-infected T-cells. The dashed lines indicate no CTL killing. Lysed cells are represented by misshapen, dashed lines. Cell types are indicated in the figure. D26-infected CD4^+^ T-cells expressing balanced levels of p12 and p8 (A); 12KO-infected CD4^+^ T-cells expressing neither p12 nor p8 (B); N26-infected CD4^+^ T-cells expressing mainly p8 (C); and G29S-infected CD4^+^ T-cells expressing mainly p12 (D).

Over-expression studies showed that p12 contributes to evasion from CTL by interacting with the MHC-class-I Heavy chain (Hc) in the ER and preventing its association with β_2_-microglobulin [27], [28], [36]. This interaction induces the MHC-class-I Hc retro-translocation into the cytosol for degradation by the proteasome, decreasing cell surface MHC-class-I. The p8 protein was recently shown in exogenous expression studies to be transferred to uninfected cells [15]. Therefore, we speculate that the contribution of p8 to CTL escape may be ascribed to the ability of this protein to be transferred to CD8^+^ T-cells, whereby it may down-regulate TCR signaling, resulting in the weakening of the strength of the immunological synapse [15], and inhibition of CTL degranulation. Indeed, the p8 protein is recruited to lipid rafts within the immunological synapse upon engagement of TCR by CD3 ligation and causes T-cell anergy [12]. More recent studies demonstrated a reduction in the strength of the immunological synapse in the presence of p8 [15]. This is in line with the finding that not only the number of HTLV-1-specific CTLs is important, but also their functional avidity [37] and even if they are abundant [38]–[40], they do not clear infection. Collectively our results suggest a model whereby a combination of effects of p8 and p12 on monocyte infectivity, viral transmission, and escape from CTL favors viral persistence (Figure 6).

A virus expressing both p12 and p8 (D26) infects monocytes, is efficiently transmitted to CD4^+^ T-cells, renders them less prone to CTL lysis and persists (Figure 6A). In contrast, a virus ablated in p8 and p12 expression (12KO) is poorly infectious in monocytes and CD4^+^T-cells *in vitro*, the infected cells are susceptible to CTL killing and infection is not sustained *in vivo* (Figure 6B). Virus expressing mainly p8 (N26), has an intermediate phenotype; it maintains its infectiousness for monocytes and CD4^+^ T-cells, but because it only partially protects infected CD4^+^ T-cells from CTL does not cause a robust infection *in vivo* (Figure 6C). Consistent with the concept of co-dependence of p8 and p12 functions for viral persistence in the host, a virus expressing mainly p12 (G29S) is poorly infectious in monocytes, the CD4^+^ infected T-cells are partially susceptible to CTL killing and the virus is not infectious in macaques (Figure 6D and Figure 3).

It is likely that p12 and p8 also affect other steps in antigen processing and presentation of HTLV-1 peptides on MHC-class-I. The p12/p8 proteins interact with calnexin and calreticulin [30] which may affect the folding of MHC-class-I and its peptide loading [41]. Similarly, the interaction of p12 and p8 with the 16 kDa protein of the V-ATPase that occurs in the ER [42] may prevent the assembly of the mature form of the V-ATPase and acidification of the secretory pathway. Recent work shows that the p8 protein traffics to the cell surface via the secretory pathway [12]. Thus, p8's association with the V-ATPase could alter not only the secretory and the endocytic pathway, but also receptor recycling on the cell membrane. Thus, future work is necessary to assess whether p12 and p8 interaction with the V-ATPase has important functional implication in antigen processing and presentation.

Our results from the macaque studies and CTL killing assays suggest that HTLV-1 virus expressing p12 only should be efficiently eliminated. However, we do find infected individuals, both carriers and HAM/TSP patients, harboring HTLV-1-p12 only virus. Several factors may influence the persistence of HTLV-1 p12 only virus. First, from earlier studies by Nicot et al [8], p12 expression through its activation of STAT5 decreases the IL-2 requirement and thus confers a proliferative advantage to infected cells. Second, we see that expression of p12 alone does down-modulate MHC class 1 expression on infected T-cells. The down-modulation could be sufficient *in vivo* to allow escape from some CTL clones. Third, p12 protein has been shown to down-modulate ICAM-1 and ICAM-2 suggesting that infected cells would be less susceptible to NK cell killing [11]. Further, while we find that HTLV-1 G29S virus does not productively infect THP-1 cells, infection does occur and in preliminary studies we find that activation of infected cells stimulates infectious virus production. This would allow the virus to persist undetected in an infected individual and upon activation spread of the virus. Finally, from our studies on p13 and Tax [43] we find that there is significant interplay between viral proteins. Our studies have focused on *orf-I* mutations, but it is possible that changes in other viral genes can impact the role of *orf-I* in immune evasion.

In conclusion, our data suggest that while infection of monocytes is important in HTLV-1 infection, viral persistence also necessitates a coordinated expression of p12 and p8 to avoid CTL recognition of infected cells. Thus, pharmacologically altering the efficiency of cleavage of the p12 precursor could have profound effects on viral persistence, by restoring the effectiveness of the host immune response to HTLV-1 and ultimately decreasing the risk of disease development through the reduction of the number of HTLV-1 infected cells.

## Materials and Methods

### Ethics statement

This study was carried out in strict accordance with the recommendations described in the Guide for the Care and Use of Laboratory Animals of the National Institute of Health, the Office of Animal Welfare and the United States Department of Agriculture. All non-human primate work was approved by the NCI Division of Intramural Research Animal Care and Use Committees (IACUC; protocol no. 458). The animals were housed, feed, given environmental enrichment and handled in accordance with the standards of the Association for the Assessment and Accreditation of Laboratory Animal Care International. Appropriate steps were taken to minimize suffering in accordance with the Weatherall report (“The use of non-human primates in research”). The animals were housed and experiments conducted at Advance Bioscience Laboratories in Rockville, MD in accordance with the standards of the American Association for Accreditation of Laboratory Animal Care. Non-human primates are housed in a rolling rack system and the cage configuration within the rooms allow for establishment of visual contact with other species members. Positive human interaction with the staff includes providing food treats, positive verbal and non-verbal communication, systematic husbandry and consistent staffing. A dietary enrichment and novel food program has been in place in the colony since 1987. Each animal is provided with sensory and cognitive enrichment that include foraging and food-based enrichment strategies, toys, auditory and visual enrichment and hideaways. All procedures were carried out under anesthesia (Telazol, Ketamine/Xylazine or Ketamine HCl) by trained personnel under the supervision of veterinary staff and all efforts were made to ameliorate the welfare and to minimize animal suffering in accordance with the Weatherall report for the use of non-human primates recommendations. Early endpoint criteria, as specified by the IACUC approved score parameters, were used to determine when animals should be humanely euthanized.

Blood samples from HTLV-1-infected patients and non-infected (ND) donors were obtained from the Centre Hospitalier Universitaire de Fort-de-France in Martinique and Institut Pasteur de Cayenne in French Guyana, the Bahia School of Medicine and Public Health and the National Institutes of Health Clinical Center. Patients suffering from HAM/TSP or HTLV-1 asymptomatic carriers were recruited according to World Health Organization (WHO) criteria. All subjects gave fully informed, written consent and all clinical investigations have been conducted according to the principles expressed in the Declaration of Helsinik. All samples were anonymized and research conformed to the guidelines of the ethics review board of the National Cancer Institute.

### Patient samples and HTLV-1 viral DNA loads

The study comprised 160 HTLV-1 infected individuals from different geographical regions (Caribbean, France, North America, Africa, and Brazil) with different disease status (Table 1). The subjects for the analysis were participants in research studies conducted at the institutions of the authors. Informed consent was written and obtained from each subject in accordance with the Declaration of Helsinki. DNA extracted from PBMCs of HTLV-1 infected individuals was used to determine the viral DNA load. Real-time PCR analysis of HTLV-1 (Tax) was performed with 100 ng of cellular DNA as previously described [19]. HTLV-1 viral DNA levels were calculated by the following formula: (copies of HTLV-1 (pX)/(copies of beta-actin/2)×100 cells. We are using the term viral DNA load since our assay does not distinguish between integrated and unintegrated viral DNA. The same DNA was used as templates for PCR reactions using Platinum High Fidelity PCR Supermix (Invitrogen, Carlsbad, CA) according to the manufacturer's protocol. In the reaction, 10 pmol/µl of each primer: 12-Fwd 5′-CACCTCGCCTTCCAACTG-3′, p12-p30-Rev 5′-GGAGTATTTGCGCATGGCC-3′ were used for amplification of the p12-p30 (872 bp) region at Tm = 55°C. For samples with no visible amplified PCR product 2 µl of the PCR reaction was used as a template for nested PCR with primers: p12-nested-F 5′-GTCTAGTATAGCCATCAACC-3′ and p30-mid-nested-Rev 5′- CTGGACAGGTGGCCAGTA-3′. PCR products were purified by gel electrophoresis and QIAquick Gel Extraction Kit (Qiagen, Valencia, CA) and subsequently, cloned into pCR4 TA TOPO vector (Invitrogen, Carlsbad, CA) according to the manufacturer's protocol. QIAprep Spin Miniprep Kit (Qiagen, Valencia, CA) was used for plasmids isolation. Five to 20 clones per patient were isolated and sequenced. The 1530 orf-I sequences for the HTLV-1 infected individuals are available from Genbank under the accession numbers in Text S1. The study on the immunophenotype of blood monocytes was performed on patient samples obtained through the NIH Clinical Center (Table 1).

### Expression plasmids

The pME18S p12deltaSL expression plasmid has been described previously [14]. This plasmid served as a backbone for generation of p12 mutants by means of PCR or by QuickChange Site-Directed Mutagenesis Kit (Stratagene, La Jolla, CA) using site-specific mutagenic oligonucleotides according to the manufacturer's instructions. The following oligonucleotides were used and the sequence of plasmid clones was analyzed to confirm the mutations.

F3L-F: 5′-CCTAGCACTATGCTGCTTCGCCTTCTCAGCfCCCT-3′

F3L-R: 5′-AGGGGCTGAGAAGGCGAAGCAGCATAGTGCTAGG-3′

S23P-F: 5′-GCTCCTGCTCTTCCTGCTTCCTCCGGGCGACGTCAGCG-3′

S23P-R: 5′-CGCTGACGTCGCCCGGAGGAAGCAGGAAGAGCAGGAGC-3′

D26N-F: 5′-CCTGCTTTCTCCGGGCAACGTCAGCGGCCTTC-3′ (for p12 subgroup A template – with S (serine) at the 23^rd^ amino acid position in p12)

D26N-R: 5′-GAAGGCCGCTGACGTTGCCCGGAGAAAGCAGG-3′ (for p12 subgroup A template)

D26N-F: 5′ -CCTGCTTCCTCCGGGCAACGTCAGCGGCCTTC-3′ (for p12 subtype B template - with P (proline) at the 23^rd^ amino acid position in p12)

D26N-R: 5′-GAAGGCCGCTGACGTTGCCCGGAGGAAGCAGG-3′ (for p12 subtype B template)

D26E-F: 5′-CTGCTTTCTCCGGGCGAAGTCAGCGGCCTTCTTC-3′

D26E-R: 5′- GAAGAAGGCCGCTGACTTCGCCCGGAGAAAGCAG-3′

G29S-F: 5′-TGCTTTCTCCGGGCGACGTCAGCAGCCTTCTTCTC-3′

G29S-R: 5′-GCGGAGAAGAAGGCTGCTGACGTCGCC-3′

delta29-F: 5′-GTGGCTCGAGACCATGCTTCTTCTCCGCCCGCCTC-3′

delta29-R: 5′-TCGGTCTAGAAACAACAACAATTGCATTCATTTTATGTTTCAGGTTCA-3′

P34L-F: 5′-GGCCTTCTTCTCCGCCTGCCTCCTGCGCCGTGC-3′

P34L-R: 5′-GCACGGCGCAGGAGGCAGGCGGAGAAGAAGGCC-3′

P45L-F: 5′-GCCTTCTCCTCTTCCTTCTTTTTCAAATACTCAGC-3′

P45L-R: 5′-GCTGAGTATTTGAAAAAGAAGGAAGAGGAGAAGGC-3′

S63P-F: 5′-CTCCCGCTCTTTTTTCCGCTTCCTCTTCTCCTC-3′

S63P-R: 5′-GAGGAGAAGAGGAAGCGGAAAAAAGAGCGGGAG-3′

L66P-F: 5′-GCTCTTTTTTTCGCTTCCTCCTCTCCTCAGCCCGTCGCTGCCG-3′

L66P-R: 5′-CGGCAGCGACGGGCTGAGGAGAGGAGGAAGCGAAAAAAAGAGC-3′

S69G-F: 5′-GCTTCCTCTTCTCCTCGGCCCGTCGCTGCCGAT-3′

S69G-R: 5′-ATCGGCAGCGACGGGCCGAGGAGAAGAGGAAGC-3′

R88K-F: 5′-GGCTCTTTCTCCCCTGGAAGGCCCCGTCGCAGCCGGCCG-3′

R88K-R: 5′-CGGCCGGCTGCGACGGGGCCTTCCAGGGGAGAAAGAGCC-3′

S91P-F: 5′-CCCCTGGAGGGCCCCGCCGCAGCCGGCCGCGGC-3′

S91P-R: 5′-GCCGCGGCCGGCTGCGGCGGGGCCCTCCAGGGG-3′. Expression of all mutants were assessed by western blot analysis using the anti-HA1 antibody clones12CA5 and 3F10-HRP (Roche Applied Science, Indianapolis, IN).

### Cell culture and DNA transfection

293T- and BHK1E6 cells were grown in Dulbecco's modified Eagle medium (DMEM) supplemented with 10% fetal bovine serum (FBS), 2 mM penicillin-streptomycin and 5 mM L-glutamine. The 729.6 B-cells were grown in RPMI 1640 supplemented with 10% FBS, 2 mM penicillin-streptomycin and 5 mM L-glutamine. The HTLV-1 molecular clones pAB-D26 (WT), pAB-G29S (p12), and pAB-p12KO were previously described. To generate pAB-N26 (p8), mutation of GAC to AAC at amino acid 26 of *orf-I* (a glutamic acid to asparagine substitution) was introduced into the pBST *Cla*I/*Sal*I cassette using QuickChange Site-Directed Mutagenesis Kit (Stratagene, La Jolla, CA) and then ligated to the pACH backbone. The mutant clones were verified by DNA sequencing of the *Cla*I/*Sal*I fragment inserted in the provirus.

To confirm that the clones were producing virus, they were transfected into 293T-cells using Effectene reagent (Qiagen, Valencia, CA). Briefly, 10 µg of DNA of pAB-D26 (WT), pAB-N26, pAB-G29S, and pAB-p12KO was transfected into 10 cm dish of 293T-cells. After 48 hours, the cells were extracted for total protein with radioimmunoprecipitation assay (RIPA) buffer and analysis of intracellular HTLV-1-p24 (Advanced BioScience Laboratories Inc., Rockville, MD) and tubulin (Sigma-Aldrich, St. Louis, MO).

Intracellular Tax expression was characterized by co-transfecting molecular clones and an HTLV-1-LTR-luciferase reporter into 293T-cells. The pRL-TKLuc plasmid was used as a transfection control. After 48 hours, cells were extracted with Passive Lysis Buffer (Promega, Milwaukee, WI) and protein samples analyzed with Dual-Glo reagent (Promega, Milwaukee, WI) for LTR activation. The culture supernatant from these transfections were spun down to remove any cell debris and analyzed by p19Gag ELISA (ZeptoMetrix, Buffalo, NY) for virus production.

The siRNA nucleofection assays were performed using the Human T-cell Nucleofection Kit (Lonza, Basel, Switzerland) and program O-017 as described by the manufacturer. Briefly, CD4^+^ D26 producing T-cells (2×10^6^) were incubated with 20 nM of either control siRNA or siRNA to *orf-I* (5′GCACUAUGCUGUUUCGCCUUCUCAG3′) (Stealth RNA, Invitrogen, Carlsbad, CA). Forty-eight hours after nucleofection, cells were used in the cytotoxicity assay. Knockdown of Orf-I expression was monitored by transient transfection of Orf-I expression constructs and siRNA into 293T-cells using Lipofectamine 2000 (Invitrogen, Carlsbad, CA) as described by the manufacturer.

### Generation and characterization of HTLV-1 producing cell lines

Stable HTLV-1 producing 729.6 human lymphoblastoid B-cells were produced as described previously [18]. Briefly 729.6 cells (5×10^6^) were electroporated with 5 µg of pAB-D26, pAB-G29S, pAB-N26 or pAB-p12KO using AMAXA Nucleofector II, Nucleofection kit V at M-013 (Lonza, Basel, Switzerland) according to the manufacturer's guidelines. Infected cells were selected by culture in neomycin as previously described [44]. The supernatant p19Gag production was measured by ELISA assay (ZeptoMetrix, Buffalo, NY). To analyze the clonality of the cell lines, 2–5×10^7^ cells were grown up in the absence of G418 and genomic DNA extracted using a Genomic DNA Wizard Kit (Promega, Milwaukee, WI) following the manufacturer's guidelines. DNA (25 µg) was digested with either High Fidelity *EcoRI* or *XhoI* (New England Biolabs, Ipswich, MA), run on an 0.8% agarose gel overnight, denatured with Denaturing Solution (Biosource, Madison, WI) and neutralized with Neutralizing Buffer (KD Medical, Columbia, MD), then blotted overnight to Immobilon NY+ membrane and crosslinked at 1.5×10^6^ J with a UV crosslinker. Biotinylated probe was synthesized with a Phototope kit and pAB-D26 molecular clone (New England Biolabs, Ipswich, MA). Membrane was prehybridized in Ultrahyb buffer (Ambion Life Technologies, Grand Island, NY) at 42°C for 2 hours, 10 pmol of probe added and hybridized overnight. Membrane was washed according to the Ultrahyb manufacturer guidelines and developed using the Phototope Star chemiluminescence kit (New England Biolabs, Ipswich, MA).

Using negative selection beads (Invitrogen, Carlsbad, CA), CD4^+^ T-cells were isolated from un-infected peripheral blood mononuclear cells. Stable HTLV-1 producing CD4^+^ T-cell lines were established by co-cultivation of donor un-infected primary HLA.A2^+^/CD4^+^ T-cells with lethally γ-irradiated 729.6-HTLV-1 infected lines. T-cells were cultured in RPMI supplemented with 20% FBS and 100 U of interleukin-2 for one year. Virus production was monitored by p19Gag ELISA (ZeptoMetrix, Buffalo, NY) and viral genomic sequences verified by sequencing of the *ClaI*-*SalI* fragment as described above.

Chronically infected THP-1 cells were produced as previously described [25]. Briefly, supernatant from 729.6 HTLV-1 producer cell lines were collected and ultra-centrifuged at 23000 rpm for two hours and thirty minutes at 4°C to concentrate the virus. Pellets were suspended in PBS and p19Gag measured by ELISA assay (ZeptoMetrix, Buffalo, NY). Equivalent amounts of p19Gag were used for infection of THP-1 cells. Briefly, THP-1 cells were suspended in virus preparations and centrifuged at 3000 rpm for one hour at room temperature in the presence of 8 µg/ml polybrene (Sigma, St. Louis, MO). Cultures were maintained in RPMI 1640, 10% FBS, with 50 µM β-mercaptoethanol and p19Gag production monitored.

### Infectivity assays

BHK1E6 cells (1×10^5^) containing a lacZ reporter gene downstream of the,LTR promoter [23], were co-cultured for 48 hours with either control un-infected cells or HTLV-1 producers D26, N26, G29S and 12KO (1×10^6^). Monolayers were washed twice with PBS to remove medium and suspension cells and assayed using a β-galactosidase Staining Kit according to manufacturer's instructions (Active Motif, Carlsbad, CA). The β-galactosidase expressing cells were counted by brightfield microscopy.

### Animal inoculation and sample collection

The macaques used in this study were male colony-bred Indian Rhesus Macaques (RMs) obtained from Covance Research Products. The animals were housed, feed, given environmental enrichment and handled in accordance with the standards of the Association for the Assessment and Accreditation of Laboratory Animal Care International. Appropriate steps were taken to minimize suffering in accordance with the Weatherall report (“The use of non-human primates in research”). The care and use of the animals were in compliance with all relevant institutional (National Institutes of Health) guidelines. All macaques were 2–3 years of age and seronegative for simian T-cell lymphotropic virus 1 and simian immunodeficiency virus at the initiation of the study. RMs were inoculated with lethally irradiated 729.6 producer cells. Supernatant p19 levels were measured prior to inoculation and cell numbers were adjusted to give equivalent amounts of p19Gag per animal (Supplementary Table S1). Four macaques each were used for the N26 and D26 viruses and eight each for the G29S virus. One macaque was infused with irradiated control parental 729.6 cells. All macaques received an equivalent dose of virus based on p19Gag expression levels per million cells, as previously described [18].

Mononuclear cells were separated from whole blood specimens by density gradient centrifugation (Ficoll). PBMCs (5×10^6^) from animals positive for proviruses were washed in PBS and DNA isolated using the Genomic DNA Wizard kit as described by the manufacturer (Promega, Milwaukee, WI).

### HTLV serology and quantitative PCR

Reactivity to specific viral antigens in the sera of infected animals was detected with the use of a commercial HTLV-1 western immunoblot assay (GeneLabs Diagnostics, Redwood City, CA). Quantitative Real-time PCR analysis was performed as described previously [18]. Proviral loads were normalized to the macaque albumin gene and expressed as the number of HTLV-1 proviral DNA copies per 10^6^ PBMCs. The limit of detection for the PCR assay is one copy in ten thousand cells. DNA sequencing of the *orf-I* genes was performed from *ex vivo* samples of macaque cellular DNA to check for reversions of point mutations to wild-type. After genomic DNA was isolated from 5×10^6^ PBMCs, PCR was performed using primers p12-Fwd, 5′-CACCTCGCCTCCCAACTG-3′ and p30-Rev, 5′-GGAGTATTTGCG- CATGGCC-3′ which amplified the fragment (871 nucleotides) spanning positions 6414 to 7285 of the HTLV-1 genome. The PCR amplicon was cloned into the pCR4TOPO vector (Invitrogen, Carlsbad, CA) using the manufacturer's protocol and 10 unique colonies sequenced. The level of orf-I mRNA was quantified using splice site-specific quantitative RT-PCR (qRT-PCR) as described [45]. After isolation of total cellular RNA, RT-PCR was performed using primers: p12-1B, 5′-GTCCGCCGTCTAG∧CACTATG-3′; p12 reverse, 5′-GGAGGAAGCAGGAAGAGC-3′; probe, MP-1 5′(FAM)-TTCGCCTTCTCAGCCCCTTGTCT-3′(TAMRA). All samples were normalized to Gapdh mRNA copy number.

### Flow cytometry

Surface staining for CD4^+^ T-cells was performed for 30 minutes at room temperature with antibodies to CD4 and HLA.A2 from BD Biosciences (San Jose, CA). All cells were fixed with 1% paraformaldehyde and at least 50,000 events acquired on an LSRII (BD Bioscience, San Jose, CA). Data analysis was performed with FlowJo 9.4 software (Tree Star Inc., Ashland, OR). THP-1 staining was performed with antibodies to CD14, CD83, HLA-DR from Biolegend (San Diego, CA) and CD80/CD86 and CCR7 from BD Bioscience (San Jose, CA).

### Cytotoxicity assay

The cytolytic activity against target cells was assayed using previously characterized HTLV-1 Tax11-19 (LLFGYPVYV)-specific CD8^+^ CTL clone [29]. The CTL clone was maintained by weekly stimulation with peptide-pulsed (1 mg/ml) irradiated PBMCs from an HLA-A201^+^ non-HTLV-1 infected individual. CTL culture medium was IMDM containing 10% human serum, 2 mM L-glutamine, 100 U/ml penicillin and 100 µg/ml streptomycin. Human recombinant interleukin-2 (50 U/ml) was added on the next day of stimulation. Target cells were CD4^+^ T-cells infected with HTLV-1 D26, N26, G29S or 12KO and autologous Epstein-Barr virus-transformed B-cells as a positive control. The cytotoxicity assay was performed using DELFIA EuTDA Cytotoxicity assay (Perkin Elmer). Target T-cells were loaded with bis (acetoxymethyl) 2,2′:6′,2″-terpyridine-6,6″-dicarboxylase (BATDA) and pulsed with or without 100 ng/ml of Tax peptide. Target cells (3×10^3^) were incubated with CTL clones for 3 hours at 37°C in 96 well plates at indicated effector-to-target ratios. The supernatant (20 µl) was incubated with 200 µl of Europium solution, and the fluorescence was measured in a fluorometer (Wallac 1420 VICTOR^3^; Perkin Elmer). The percent specific lysis was calculated as (experimental release−spontaneous release)/(maximum release−spontaneous release)×100. The assay was performed in triplicate.

## Supporting Information

Table S1 Extracellular p19Gag production and viral DNA copy in HTLV-1 producer B-cells.
(DOCX)

Table S2 Characterization of HTLV-1 infected CD4^+^ T-cells.
(DOCX)

Table S3 Viral DNA loads for patients with G29S mutations.
(DOCX)

Text S1 Genbank accession numbers for orf-I sequences from HTLV-1 infected individuals.
(TXT)
