# Supplementary material for: Co-dependence of HTLV-1 p12 and p8 Functions in Virus Persistence
Source: PLoS Pathog. 2014 Nov 6;10(11):e1004454. doi: 10.1371/journal.ppat.1004454 (PMC4223054; doi:10.1371/journal.ppat.1004454)
Supplement: Table S1 — Extracellular p19Gag production and viral DNA copy in HTLV-1 producer B-cells. (DOCX) [file ppat.1004454.s001.docx]

**Table S1.** Extracellular p19Gag production and viral DNAcopy in HTLV-1 producer B-cells.

| **Sample** | **HTLV viral DNA copy/cell** | **p19Gag (ng/ml)** |
| --- | --- | --- |
| **729.6 cell lines** | | |
| **D26** | **2.12** | **35.9** |
| **N26** | **4.21** | **39.1** |
| **G29S** | **1.42** | **15.8** |
| **p12KO** | **1.53** | **30.69** |
