# Supplementary material for: Co-dependence of HTLV-1 p12 and p8 Functions in Virus Persistence
Source: PLoS Pathog. 2014 Nov 6;10(11):e1004454. doi: 10.1371/journal.ppat.1004454 (PMC4223054; doi:10.1371/journal.ppat.1004454)
Supplement: Table S2 — Characterization of HTLV-1 infected CD4+ T-cells. (DOCX) [file ppat.1004454.s002.docx]

**Table S2.** Characterization of HTLV-1 infected CD4^+^ T-cells.

| **Sample** | **HTLV viral DNA copy/cell** | **p19Gag (ng/ml)** | **orf-I mRNA copy number/gapdh copy number** |
| --- | --- | --- | --- |
| **CD4^+^ cell lines** | | |  |
| **D26** | **1.29** | **90.1** | **1892.7** |
| **N26** | **1.69** | **52.9** | **930.4** |
| **G29S** | **2.49** | **50.4** | **5089.9** |
| **p12KO** | **1.21** | **69.5** | **268.6** |
