# Supplementary material for: Co-dependence of HTLV-1 p12 and p8 Functions in Virus Persistence
Source: PLoS Pathog. 2014 Nov 6;10(11):e1004454. doi: 10.1371/journal.ppat.1004454 (PMC4223054; doi:10.1371/journal.ppat.1004454)
Supplement: Table S3 — Viral DNA loads for patients with G29S mutations. (DOCX) [file ppat.1004454.s003.docx]

**Table S3.** Viral DNA loads for patients with G29S mutations.

| **Sample** | **Viral DNA Load**  **(%)** | **Disease Status** |
| --- | --- | --- |
|  | | |
| **South America/Brazil** | **3.11**  **0.55**  **1.83**  **0.21**  **8.536**  **0.0415** | **Carrier**  **Carrier**  **Carrier**  **Carrier**  **Carrier**  **Carrier** |
| **Africa** | **17.63**  **11.94** | **Carrier**  **Carrier** |
| **Caribbean**  **North America** | **11.21**  **10.8**  **7.5**  **34.28**  **17.48** | **Carrier**  **Carrier**  **Carrier**  **HAM/TSP**  **HAM/TSP** |
| **Unknown** | **94.8**  **6.47**  **1.82**  **35.54** | **HAM/TSP**  **HAM/TSP**  **HAM/TSP**  **HAM/TSP** |
